# Performance and cross-crop resistance of Cry1F-maize selected *Spodoptera frugiperda* on transgenic Bt cotton: implications for resistance management

**DOI:** 10.1038/srep28059

**Published:** 2016-06-15

**Authors:** Fei Yang, David L. Kerns, Sebe Brown, Ryan Kurtz, Tim Dennehy, Bo Braxton, Graham Head, Fangneng Huang

**Affiliations:** 1 Macon Ridge Research Station, Louisiana State University Agricultural Center, Winnsboro, LA 71295, USA; 2 Department of Entomology, Louisiana State University Agricultural Center, Baton Rouge, LA 70803, USA; 3 Cotton Incorporated, 6399 Weston Parkway, Cary, NC 27513, USA; 4 Bayer CropScience, Research Triangle Park, NC 27709, USA; 5 Dow AgroSciences, Travelers Rest, SC, 29690, USA; 6 Monsanto Company, St. Louis, MO 63167, USA

## Abstract

Transgenic crops producing *Bacillus thuringiensis* (Bt) proteins have become a primary tool in pest management. Due to the intensive use of Bt crops, resistance of the fall armyworm, *Spodoptera frugiperda*, to Cry1F maize has occurred in Puerto Rico, Brazil, and some areas of the southeastern U.S. The sustainability of Bt crops faces a great challenge because the Cry1F-maize resistant *S. frugiperda* may also infest other Bt crops in multiple cropping ecosystems. Here we examined the survival and plant injury of a *S. frugiperda* population selected with Cry1F maize on three single-gene and five pyramided Bt cotton products. Larvae of Cry1F-susceptible (SS), -heterozygous (RS), and -resistant (RR) genotypes of *S. frugiperda* were all susceptible to the pyramided cotton containing Cry1Ac/Cry2Ab, Cry1Ac/Cry1F/Vip3A, Cry1Ab/Cry2Ae, or Cry1Ab/Cry2Ae/Vip3A, and the single-gene Cry2Ae cotton. Pyramided cotton containing Cry1Ac/Cry1F was effective against SS and RS, but not for RR. These findings show that the Cry1F-maize selected *S. frugiperda* can cause cross-crop resistance to other Bt crops expressing similar insecticidal proteins. Resistance management and pest management programs that utilize diversify mortality factors must be implemented to ensure the sustainability of Bt crops. This is especially important in areas where resistance to single-gene Bt crops is already widespread.

Since 1996, transgenic crops expressing *Bacillus thuringiensis* (Bt) proteins have been widely planted in many countries^1^. Currently, maize, cotton and soybean are the three major commercial Bt crops. In 2014 alone, a total of >77 million hectares of Bt crops were planted worldwide, consisting of 62.4% maize, 31.1% cotton and 6.5% soybean^1^. These Bt crops are effective in controlling their target insect pests while causing little or no harm to non-target organisms^2^,^3^. Bt crops have offered great benefits including reduced chemical insecticide use and crop yield loss^2^,^3^,^4^,^5^,^6^,^7^. However, the extensive use of Bt crops has placed a strong selection pressure on target pest populations, with the potential consequence of rapid evolution of resistance, threatening these benefits^8^,^9^,^10^. In recent years, field-evolved resistance to Bt crops that resulted in control problems has occurred in some target pests^11^,^12^,^13^,^14^,^15^,^16^.

The fall armyworm, *Spodoptera frugiperda*, is a well-known long-distance migratory insect native to the tropical regions of the Western Hemisphere from the U.S. to Argentina^17^. Field resistance to Cry1F maize in *S. frugiperda*, due to the intensive use of Bt crops, has been documented in Puerto Rico^11^, Brazil^15^, and the U.S. mainland^16^. To date, *S. frugiperda* is the first and only target pest to develop field resistance to Bt crops, at multiple locations, across different countries and continents. Currently, all the three major Bt crops are planted in Brazil throughout the year^18^, while in the southern U.S., Bt maize and Bt cotton are planted in close proximity to one another^16^. Cry1F protein is expressed in many varieties of these Bt maize and Bt cotton currently planted in the U.S. and Brazil. *S. frugiperda* is a cross-crop target of the Bt maize and Bt cotton in both countries, as well as, a secondary target of Bt soybean in Brazil. Thus, there is a great concern that Cry1F-maize resistant *S. frugiperda* may also infest other Bt crops where multiple Bt crops are planted in the landscape.

Available information shows that the genetic elements (e.g. transgenes and promoters) used in producing Bt crops can be different from crop to crop. For example, the Cry1F gene in the cotton plants (event DAS-24236-5) is a synthetic, plant-optimized, full length version of Cry1F gene, while it is a plant-optimized version of truncated Cry1F gene in the maize plants (TC1507)^19^. The promoter used in Cry1F maize is an ubiquitin promoter, but in Cry1F cotton it is a mannopine synthase promoter^19^. In addition, the expression level of a Bt gene could vary considerably depending on crops, plant stages, and plant parts^19^,^20^,^21^,^22^. The toxicity of Bt crops can also be affected by plant-Bt gene interactions^23^,^24^,^25^. For instance, some plant secondary compounds can alter the toxicity of Bt proteins against lepidopteran larvae, either positively or negatively^23^,^24^,^25^. Thus, the resistance level and the related cross-resistance pattern of a pest population could be distinct for different Bt crops. As a result, an insect population that has adapted to Cry1F maize plants may not be resistant to Cry1F cotton. For this reason, here we introduce a term ‘cross-crop resistance’, referring that a resistant insect population selected with one Bt crop is also resistant to other crops containing similar or different Bt genes. In multiple Bt cropping ecosystems, understanding the patterns of cross-crop resistance is important for crop layout to confine the resistance spread.

Numerous studies have documented that cross-resistance frequently occurs among closely-related Bt proteins and insect species^26^,^27^,^28^,^29^,^30^. However, all of the previous studies involved comparisons of resistance that was selected with one Bt protein to cross resistance to other Bt proteins either using diet bioassays or plant products of the same crop. To date, there has no information available about cross-crop resistance among Bt crops for any target species. Here we used a highly Cry1F- resistant strain of *S. frugiperda* selected on Cry1F maize to assess the cross-crop resistance to Bt cotton. We evaluated the survival, growth, development, and plant injury of the Cry1F maize-susceptible (SS), -heterozygous (RS), and -resistant (RR) genotypes of *S. frugiperda* on one non-Bt, three single-Bt gene and five pyramided Bt cotton products. We found that Cry1F-maize resistant *S. frugiperda* was also highly resistant to WideStrike cotton expressing the Cry1Ac and Cry1F proteins. The results here provide the first experimental evidence that resistance to Bt proteins selected with one Bt crop can result in resistance to other crops containing similar Bt proteins.

## Results

**Figure 1:**
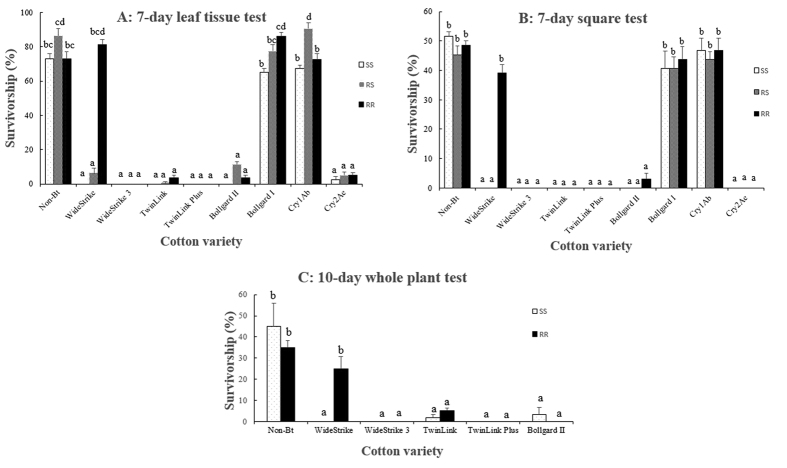
Survivorship of Cry1F-susceptible (SS), -heterozygous (RS), and -resistant (RR) genotypes of *S. frugiperda* on leaf tissues (**A**), squares (**B**) and whole plants (**C**) of one non-Bt and eight Bt cotton varieties/lines: Bollgard I (Cry1Ac), Bollgard II (Cry1Ac/Cry2Ab), WideStrike (Cry1Ac/Cry1F), WideStrike 3 (Cry1Ac/Cry1F/Vip3A), TwinLink (Cry1Ab/Cry2Ae), TwinLink Plus (Cry1Ab/Cry2Ae/Vip3A), and two experimental lines expressing a single Bt gene of Cry1Ab and Cry2Ae, respectively. Mean values in a figure followed by a same letter are not significantly different (Tukey’s HSD test, α = 0.05).

**Figure 2:**
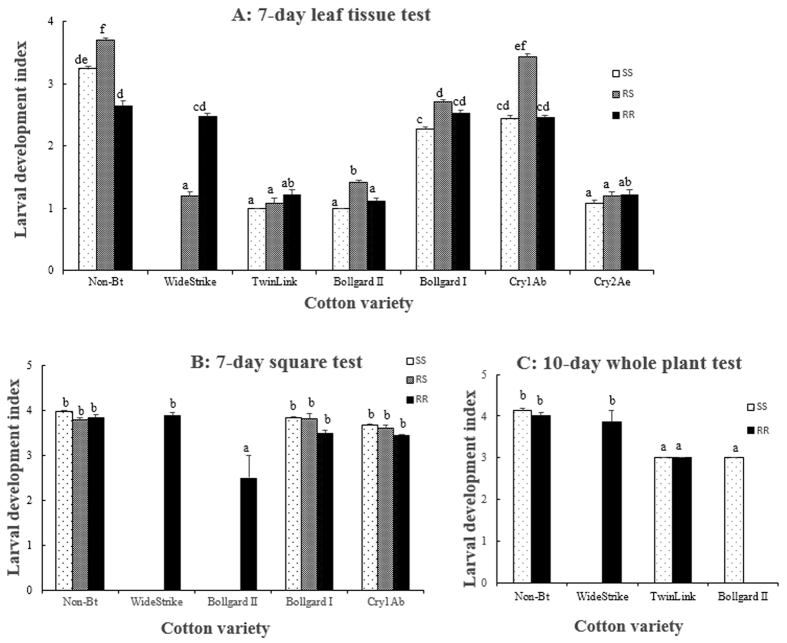
Larval development of Cry1F-susceptible (SS), -heterozygous (RS), and -resistant (RR) genotypes of *S. frugiperda* on leaf tissues (**A**), squares (**B**) and whole plants (**C**) of one non-Bt and eight Bt cotton varieties/lines: Bollgard I (Cry1Ac), Bollgard II (Cry1Ac/Cry2Ab), WideStrike (Cry1Ac/Cry1F), WideStrike 3 (Cry1Ac/Cry1F/Vip3A), TwinLink (Cry1Ab/Cry2Ae), TwinLink Plus (Cry1Ab/Cry2Ae/Vip3A), and two experimental lines expressing a single Bt gene of Cry1Ab and Cry2Ae, respectively. Mean values in a figure followed by a same letter are not significantly different (Tukey’s HSD test, α = 0.05).

Two independent leaf tissue bioassays were conducted in the laboratory to evaluate larval survival and development of the three genotypes (SS, RR, and RS) of *S. frugiperda* on a non-Bt, three single-gene and five pyramided Bt cotton products (Table S1). Results of the two bioassays were consistent. Larval survival on non-Bt leaf tissue was not different (P > 0.05) among insect genotypes with an average survivorship rate of 79.2% after 7 days (Fig. 1A). Leaf tissue of the single gene Cry1Ab or Cry1Ac cotton was not effective for controlling the three insect genotypes with a survivorship of 65.0–90.6% (Fig. 1A), but larval development of the survivors was somewhat delayed compared to that on non-Bt leaf tissue (Fig. 2A). Pyramided Bt cotton products Bollgard II (expressing Cry1Ac and Cry2Ab), TwinLink (expressing Cry1Ab and Cry2Ae), TwinLink Plus (expressing Cry1Ab, Cry2Ae and Vip3A), and WideStrike 3 (expressing Cry1Ac, Cry1F and Vip3A), and the single gene Cry2Ae cotton were effective against all three insect genotypes. Survivorship of the three genotypes on these five Bt products was low (0.0 to 11.3%), and the development of the limited survivors was severely delayed (Figs 1A,2A and S1A). Survivorship of SS and RS on leaf tissue of WideStrike cotton expressing Cry1Ac and Cry1F was also low (0% for SS and 6.3% for RS). However, 81.3% RR survived on leaf tissue of WideStrike, and most survivors were 2^nd^ and 3^rd^ instars. Both the larval survivorship and development index of RR on WideStrike were not significantly (P > 0.05) different than the larvae reared on the non-Bt cotton leaf tissue. In addition, the larval weight of RR on leaf tissue of WideStrike was also not significantly (P > 0.05) different from that on the non-Bt leaf tissue (Fig. S1A).

In the field, later instars of *S. frugiperda* usually move away from leaf tissue and feed on fruiting structures of cotton plants. To examine if the performance of *S. frugiperda* was consistent among different structures of cotton plants, we assayed the 7-day survival of the three insect genotypes on cotton squares (flower buds) of the non-Bt and eight Bt cotton products described above in the laboratory. As observed in the leaf tissue bioassays, larval survivorship of the three insect genotypes was not different (P > 0.05) among non-Bt, Cry1Ab and Cry1Ac squares (40.6–51.6%; Fig. 1B). Bollgard II, TwinLink, TwinLink Plus, WideStrike 3, and Cry2Ae squares were highly toxic to all three genotypes with a survivorship rate of 0.0–3.1% (Fig. 1B). No survivors of SS or RS were observed on WideStrike squares. However, 39.1% of RR larvae survived on WideStrike squares, which was not significantly (P > 0.05) different from that on the non-Bt squares (48.4%). Both larval development and body weight of RR on WideStrike squares were also not significantly (P > 0.05) different from those on the non-Bt squares (Figs 2B and S1B).

Finally, we conducted two independent greenhouse tests with second instars of *S. frugiperda* to measure larval survival, growth, development, and plant injury of the insect on whole cotton plants. Limited by the greenhouse space and the unavailability of some regulated cotton seeds, the first test evaluated only SS and RR genotypes on the non-Bt and five Bt cotton products including WideStrike, WideStrike 3, TwinLink, TwinLink Plus, and Bollgard II. The second test included all three insect genotypes and all nine cotton products listed in Table S1. In the first test, larval survival of SS and RR was not significantly (P > 0.05) different on the non-Bt cotton plants with an average of 40% survivorship after 10 days (Fig. 1C). No live larvae of SS were observed on WideStrike, WideStrike 3, and TwinLink Plus while no live larvae of RR were found on WideStrike 3, TwinLink Plus, and Bollgard II. A few SS larvae were found on TwinLink (1.7% survivorship) and Bollgard II (3.3% survivorship) plants. On TwinLink plants, 5% RR larvae were alive. However, a significant number of RR larvae survived on WideStrike plants (25.0%), which was not significantly different (P > 0.05) from that on non-Bt plants (35.0%; Fig. 1C). Larval development and body weight of RR on WideStrike plants were also not significantly (P > 0.05) different from those on the non-Bt plants (Figs 2C and S1C). Because most larvae had moved off the plants when data were recorded in the second test, data on survivorship were not available from this test.

**Figure 3:**
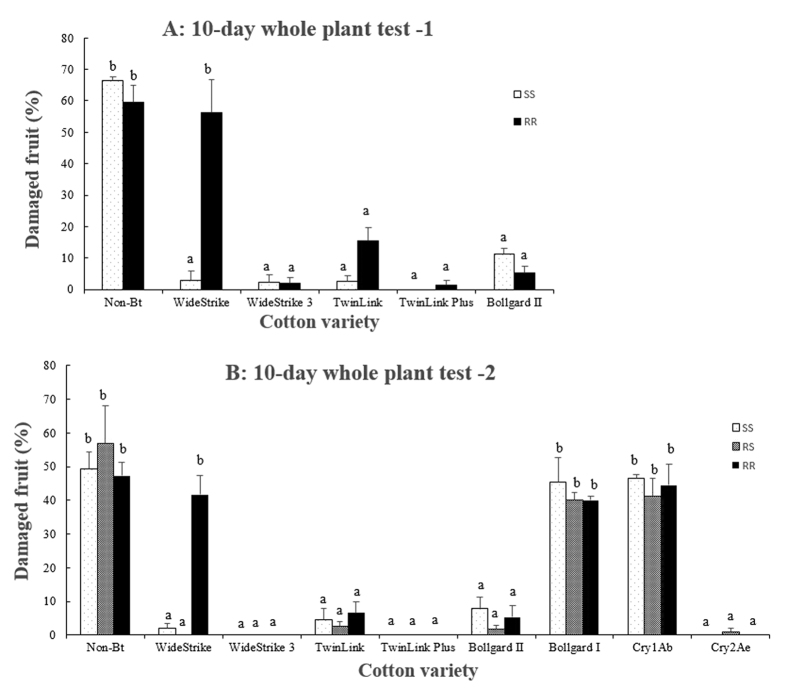
Percentage of injured fruits (square, flower, and boll) by Cry1F-susceptible (SS), -heterozygous (RS), and -resistant (RR) genotypes of *S. frugiperda* on whole plants of one non-Bt and eight Bt cotton varieties/lines: Bollgard I (Cry1Ac), Bollgard II (Cry1Ac/Cry2Ab), WideStrike (Cry1Ac/Cry1F), WideStrike 3 (Cry1Ac/Cry1F/Vip3A), TwinLink (Cry1Ab/Cry2Ae), TwinLink Plus (Cry1Ab/Cry2Ae/Vip3A), and two experimental lines expressing a single Bt gene of Cry1Ab and Cry2Ae, respectively. Mean values in a figure followed by a same letter are not significantly different (Tukey’s HSD test, α = 0.05).

In both greenhouse tests, percentages of injured fruit (squares, flowers and bolls) on non-Bt cotton were not significantly (P > 0.05) different among insect genotypes (47.3–66.3%; Fig. 3). No or little injury (0.0–2.3%) was observed on WideStrike 3, TwinLink Plus, and Cry2Ae plants for all three insect genotypes. TwinLink and Bollgard II plants showed an injury rate of 1.8–15.6%, which was not significantly (P > 0.05) different than the rate on WideStrike 3, TwinLink Plus, and Cry2Ae plants (Fig. 3). Again, Cry1Ab and Cry1Ac cotton were not effective in protecting the plants from injury by the three insect genotypes with a fruit injury rate of 40.0 to 46.4% (Fig. 3B), which were not significantly (P > 0.05) different from that on non-Bt plants. Little or no injury (0.0–3.0%) was observed on WideStrike plants infested with SS and RS. In contrast, 41.7–51.6% of WideStrike cotton fruit were injured by RR, which was not significantly (P > 0.05) different from the rate observed on non-Bt plants (Fig. 3).

## Discussion

Transgenic Bt crop technology offers great benefits for pest control^3^,^4^,^5^,^6^,^7^. However, the durability and efficacy of this technology can be diminished by resistance evolution. The current study shows that resistance resulting from selection with Cry1F-maize can result in cross-crop resistance to transgenic cotton containing the similar Bt proteins. The results also suggest that the dissimilar forms of the Cry1F gene inserted in the maize and cotton plants apparently did not change the mode of action of the Cry1F toxin. Although the demonstrated cross-crop resistance in *S. frugiperda* between Cry1F maize and WideStrike cotton is not unexpected, because both crops containing Cry1F gene, coupled with the ineffectiveness of Cry1Ac against this pest, this documentation of the cross-crop resistance in *S. frugiperda* still has important implications for resistance management. In recent years, field-evolved resistance of *S. frugiperda* to Cry1F maize has resulted in widely occurring field control problems in Puerto Rico and Brazil^11^,^15^. The same resistance problem has also been found in some areas of the southeastern U.S.^16^. As mentioned above, in the U.S. cotton belt where Bt maize and Bt cotton are planted in close proximity to one another, selection pressure and risk for resistance development are greater for cross-crop targets of *S. frugiperda*. Similar challenges can also be present in other mixed cropping systems such as in Brazil where *S. frugiperda* is a cross-crop target of Bt maize, cotton and soybean^16^,^31^,^32^. Our results suggest that if cross-crop resistance occurs among different Bt crops, landscapes consisting of multiple Bt crops such as in the U.S. cotton belt can extend the selection period for cross-crop insects and thus accelerate resistance evolution^16^,^32^.

Previous studies reported that pyramided Bt maize containing Cry2A and/or Vip3A is effective against Cry1F-resistant *S. frugiperda*^16^,^33^. Results of the current study also demonstrate that the highly resistant *S. frugiperda* selected with Bt maize is susceptible to pyramided Bt cotton expressing Cry2A and/or Vip3A. Thus pyramided Bt maize and Bt cotton containing Cry2A and/or Vip3A genes should provide a means for managing the Cry1F resistance in *S. frugiperda*. However, there is a high risk of resistance evolving to the current pyramided Bt crops in areas where resistance to Cry1F maize has widely occurred. In the current transgenic Bt crop market, Bt genes targeting lepidopteran pests in the three commercial Bt crops can be classified into three groups: Cry1 which includes Cry1Ab, Cry1Ac, Cry1F, and Cry1A.105; Cry2 which includes Cry2Ab and Cry2Ae; and Vip3A. Studies have shown that there is a high level of cross-resistance among Cry1 proteins in *S. frugiperda*^16^,^29^, and the Cry1Ab and Cry1Ac proteins are not highly active against *S. frugiperda*^33^,^34^,^35^. Thus, once resistance to Cry1F occurs, the efficacy of all currently utilized Cry1 proteins is likely to be affected, leaving only Cry2A and Vip3A as being active in the three Bt crops.

In addition, our study also showed that some susceptible and heterozygous larvae can survive on TwinLink and Bollgard II traits in the whole plant tests, suggesting that the Bt protein expression in the two pyramided Bt cotton products is likely not a “high dose”. A violation of the “high dose/refuge” resistance management strategy would further accelerate resistance development to these Bt products. Furthermore, durability of these remaining active proteins against Cry1F-resistant *S. frugiperda* is likely to be undermined by an abundance of Bt maize in which Cry2A or Vip3A is pyramided with Cry1A proteins. Recently, Santos-Amaya *et al*.^36^ conducted laboratory selections of a *S. frugiperda* strain already resistant to Cry1F maize with pyramided Bt maize expressing Cry1A.105 and Cry2Ab2 proteins. After 10 generations of selection, they produced a strain that was resistant to the pyramided Bt maize. The study by Santos-Amaya *et al*.^36^ shows how rapidly resistance to pyramided Bt crops could occur once resistance/cross-resistance to one Bt gene is present. Thus, resistance management and pest management programs that diversify mortality factors, rather than just relying on the limited modes of action of Bt toxins, are needed to ensure the sustainability of Bt crops^37^, especially in the areas where resistance to single-gene Bt crops is already widespread.

## Methods

### Insect sources and cotton products

Two laboratory strains, SS and RR, of *S. frugiperda* were used as the original insect sources in this study. SS was collected from non-Bt maize fields near Weslaco, TX in 2013. Since then, it has never been exposed to any Bt proteins or insecticides in the laboratory. SS is susceptible to both Cry1F maize plants and purified Cry1F protein in laboratory bioassays and greenhouse whole plant tests^16^,^38^. RR was developed from an F_2_ screen with two-parent families derived from feral individuals collected from non-Bt maize fields in south Florida in 2011^16^. RR is highly resistant to purified Cry1F protein (>270-fold) in diet-incorporated bioassays and whole Cry1F maize plants in the greenhouse^16^. Before the current study, RR had been backcrossed with SS twice and re-selected for resistance with Cry1F maize leaf tissue for two generations. In addition, a heterozygous genotype, RS, was developed from reciprocal crosses between RR and SS. In this study, performance of the three insect genotypes, SS, RR, and RS, were evaluated on one non-Bt and three single-gene and five pyramided cotton products representing different Bt traits (Table S1). Among these eight Bt cotton products, WideStrike, WideStrike 3, TwinLink and Bollgard II were common commercial products in the U.S., while Cry1Ab, Cry2Ae, Bollgard I, and TwinLink Plus were regulated under the U.S. Environmental Protection Agency during the trial period.

### Plant tissue bioassays

A total of three independent bioassays were performed with cotton plant tissue in the laboratory: two with leaves and one with cotton squares. Greenhouse-grown plants were used in the two-leaf tissue bioassays, while the squares used in the study were from field-grown plants. To produce the appropriate aged leaves for the bioassays, cotton plants were cultivated in 18.9-liter plastic pots filled with a standard potting soil mixture (Perfect Mix, Expert Gardener products, St. Louis, MO) in greenhouse at two locations, one at the Louisiana State University (LSU) Agricultural Center’s Macon Ridge Research Station in Winnsboro, LA and the other at the LSU Ag Center’s Central Research Station in Baton Rouge, LA. In the greenhouse plantings, two to three plants per pot were maintained with regular irrigation and fertilization for optimal growth during experiments. When plants were six to eight nodes above white flower, fully expanded leaves were collected from the plants and used in the laboratory bioassays. In the leaf tissue bioassays, one leaf of a cotton variety was placed in each well of 8-well trays (C-D International, Pitman, NJ), and ten neonates of one of the three insect genotypes were then placed on the leaf tissue in the well. In each leaf tissue bioassay, there were four replications for each combination of cotton product and insect genotype, and each replication consisted of two wells each with 10 larvae (n = 4 × 20 = 80). Bioassay trays with larvae and leaf tissue were placed in growth chambers maintained at 28 °C, 50% RH, and a 16:8 (L:D) h photoperiod. Leaves were replaced every two days. Larval survival, growth (body weight), and development were recorded on the 7^th^ day after larval infestation.

To produce the properly aged cotton squares for the square tissue bioassay, seeds of the nine cotton products mentioned above (Table S1) were planted in a field at the LSU AgCenter’s Macon Ridge Research Station in Winnsboro, LA. Squares were collected from the field and used in the laboratory bioassay. In the bioassay, two cotton squares were placed in each well of 32-well trays (C-D International, Pitman, NJ), and then two early second instars of an insect genotype were placed on the squares (1 insect/square) in the well. There were four replications for each combination of cotton product and insect genotype, and each replication contained 8 wells each with 2 larvae (n = 4 × 16 = 64). Squares were replaced every 1–2 days. Bioassay trays with square tissue were placed in growth chambers maintained under the same conditions as used in the leaf tissue bioassays. Larval survival, growth, and development was recorded on the 7^th^ day after larval release.

### Whole plant tests

Two independent tests were conducted to evaluate the performance of *S. frugiperda* on whole non-Bt and Bt cotton plants in the greenhouse. The first test was located at the LSU AgCenter’s Macon Ridge Research Station in Winnsboro and the second was conducted at the Central Research Station in Baton Rouge, LA. Both tests were initiated at the onset of the anthesis stage of the plants. A preliminary test showed that 2^nd^ instars of *S. frugiperda* on the whole plants of non-Bt cotton in the greenhouse exhibited a much better survival than the newly-hatched neonates. Therefore, 2^nd^ instars were used in the greenhouse tests. For each greenhouse test, fifteen early second instars of an insect genotype were manually placed onto the leaves of the fifth node of a cotton plant. In the first test, all larvae were confined to the plants using nylon mesh fabric cages, while no cages were used for larvae confinement in the second test. Each combination of cotton product and insect genotype was replicated four times in a randomized complete block design with one pot (2–3 plants) per replication. Larval survival, growth, and development, as well as percentage of injured fruits (flower, square and boll) were recorded on the 10^th^ day after larval release for the first test, while only fruit injury was recorded for the second test because the mature larvae had moved off the plants and dropped to the soil for pupation.

### Data analysis

Larval developmental stages were converted to a development index: 1 = 1^st^ instar, 2 = 2^nd^ instar, …, 5 = 5^th^ instar as described in Yang *et al*.^39^. Data on development index and larval body weight were transformed to the log(x + 1) scale, while larval survivorship and percentage of injured fruits were transformed using arcsine of (x^0.5^) to normalize treatment variances^40^. The transformed data were then analyzed using two-way analysis of variance with insect strain and cotton product as the two main factors^41^. Because the results of the two leaf tissue bioassays in the laboratory were consistent, data on larval survivorship, body weight, and development index for the leaf tissue bioassays were pooled for statistical analysis and the pooled data were analyzed using mixed models with bioassay as a random factor^41^. Treatment means for each test and the pooled data were separated using Tukey’s honestly significant differences at α = 0.05 level.

## Additional Information

**How to cite this article**: Yang, F. *et al*. Performance and cross-crop resistance of Cry1F-maize selected *Spodoptera frugiperda* on transgenic Bt cotton: implications for resistance management. *Sci. Rep.* **6**, 28059; doi: 10.1038/srep28059 (2016).

## Supplementary Material

Supplementary Information
