# Supplementary material for: Performance and cross-crop resistance of Cry1F-maize selected Spodoptera frugiperda on transgenic Bt cotton: implications for resistance management
Source: Sci Rep. 2016 Jun 15;6:28059. doi: 10.1038/srep28059 (PMC4908423; doi:10.1038/srep28059)
Supplement: Supplementary Information [file srep28059-s1.pdf]

Performance and cross-crop resistance of Cry1F-maize selected *Spodoptera frugiperda* on transgenic Bt cotton: implications for resistance management

Fei Yang, David L. Kerns, Sebe Brown, Ryan Kurtz, Tim Dennehy, Bo Braxton, Graham Head, & Fangneng Huang

Supplementary Information

Table S1. Cotton varieties/lines evaluated in the study.

Supplier	Designation	Variety	Bt proteins
Bayer Cropscience	Non-Bt	FM 966 LL	None
	Cry1Ab	T304-40	Cry1Ab
	Cry2Ae	GHB119	Cry2Ae
	TwinLink	FM 966 TwinLink	Cry1Ab, Cry2Ae
	TwinLink Plus	FM 966 TwinLink Plus	Cry1Ab, Cry2Ae, Vip3A
Monsanto	Bollgard I	DPL 555 BR	Cry1Ac
	Bollgard II	DP 0912 B2RF	Cry1Ac, Cry2Ab
Dow Agrosciences	WideStrike	PHY 499 WRF	Cry1Ac, Cry1F
	WideStrike 3	PHY 495 W3RF	Cry1Ac, Cry1F, Vip3A

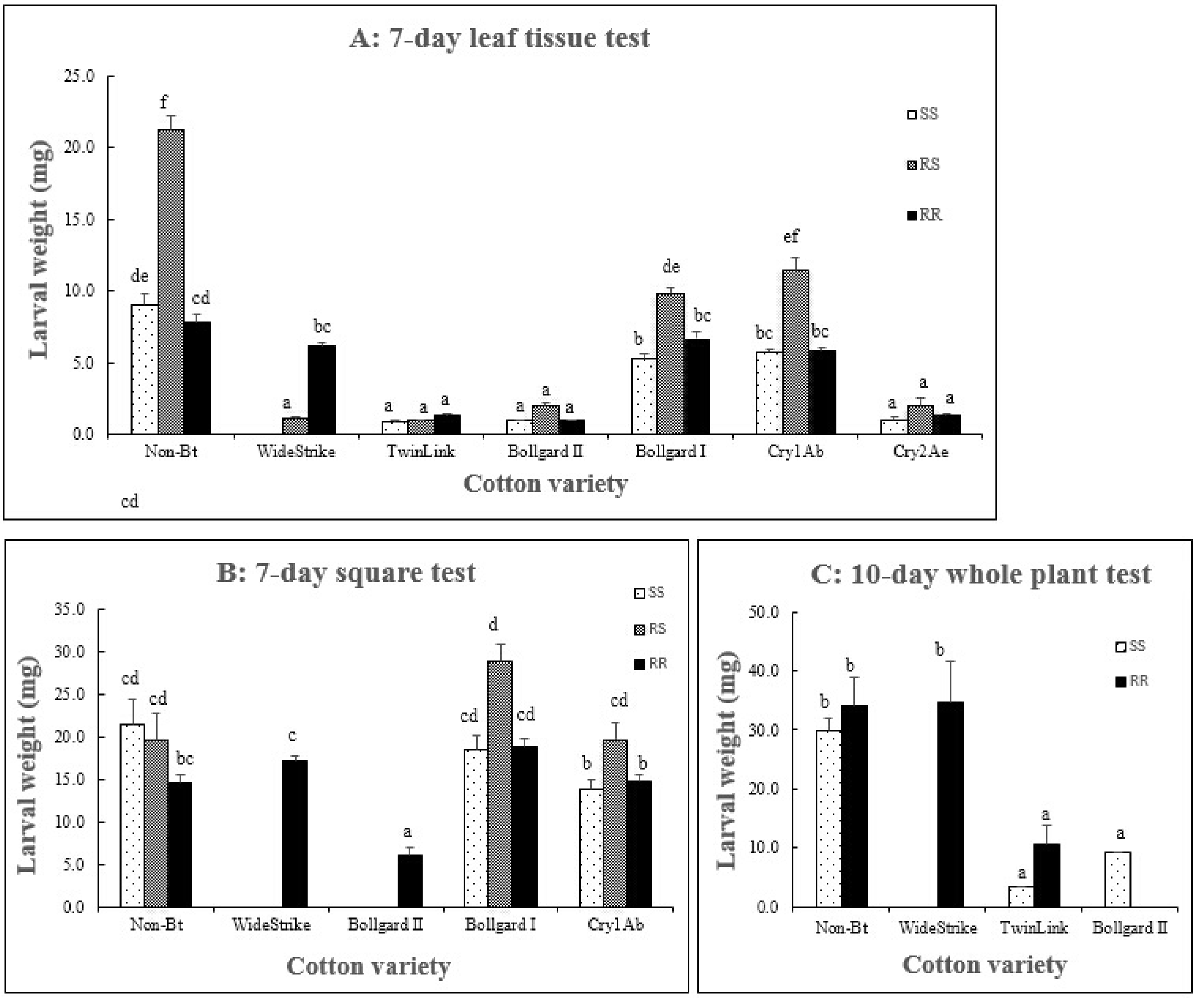

Fig. S1. Larval weight of Cry1F-susceptible (SS), -heterozygous (RS), and -resistant (RR) genotypes of *S. frugiperda* on leaf tissues (A), squares (B) and whole plants (C) of one non-Bt and eight Bt cotton varieties/lines: Bollgard I (Cry1Ac), Bollgard II (Cry1Ac/Cry2Ab), WideStrike (Cry1Ac/Cry1F), WideStrike 3 (Cry1Ac/Cry1F/Vip3A), TwinLink (Cry1Ab/Cry2Ae), TwinLink Plus (Cry1Ab/Cry2Ae/Vip3A), and two experimental lines expressing a single Bt gene of Cry1Ab and Cry2Ae, respectively. Mean values in a figure followed by a same letter are not significantly different (Tukey's HSD test, $\alpha = 0.05$).
